# A Multicenter Study of Patient-Reported Infectious and Noninfectious Complications Associated With Indwelling Urethral Catheters

**DOI:** 10.1001/jamainternmed.2018.2417

**Published:** 2018-07-02

**Authors:** Sanjay Saint, Barbara W. Trautner, Karen E. Fowler, John Colozzi, David Ratz, Erica Lescinskas, John M. Hollingsworth, Sarah L. Krein

**Affiliations:** 1 Center for Clinical Management Research, Veterans Affairs Ann Arbor Healthcare System, Ann Arbor, Michigan; 2 Department of Internal Medicine, University of Michigan Medical School, Ann Arbor; 3 Ann Arbor Veterans Affairs Medical Center/University of Michigan Patient Safety Enhancement Program, Ann Arbor; 4 Center for Innovations in Quality, Effectiveness and Safety, Michael E. DeBakey Veterans Affairs Medical Center, Houston, Texas; 5 Department of Internal Medicine, Baylor College of Medicine, Houston, Texas; 6 Department of Urology, University of Michigan Medical School, Ann Arbor

## Abstract

**Importance:**

Indwelling urethral catheters (ie, Foley catheters) are important in caring for certain hospitalized patients but can also cause complications in patients.

**Objective:**

To determine the incidence of infectious and noninfectious patient-reported complications associated with the indwelling urethral catheter.

**Design, Setting, and Participants:**

A prospective cohort study of consecutive patients with placement of a new indwelling urethral catheter while hospitalized at 1 of 4 US hospitals in 2 states. The study was conducted from August 26, 2015, to August 18, 2017. Participants were evaluated at baseline and contacted at 14 days and 30 days after insertion of the catheter about complications associated with the indwelling urethral catheter and how catheterization affected their social activities or activities of daily living.

**Exposures:**

Indwelling urethral catheter placement during hospitalization. Patients were enrolled within 3 days of catheter insertion and followed up for 30 days after catheter placement, whether the catheter remained in or was removed from the patient.

**Main Outcomes and Measures:**

Infectious and noninfectious complications associated with an indwelling urethral catheter as well as how the catheter affected patient social activities or activities of daily living.

**Results:**

Of 2967 eligible patients, 2227 (75.1%) agreed to participate at 1 of 4 study sites; 2076 total patients were evaluated. Of these, 71.4% were male; mean (SD) age was 60.8 (13.4) years. Most patients (1653 of 2076 [79.6%]) had short-term catheters placed for surgical procedures. During the 30 days after urethral catheter insertion, 1184 of 2076 patients (57.0%; 95% CI, 54.9%-59.2%) reported at least 1 complication due to the indwelling urethral catheter. Infectious complications were reported by 219 of 2076 patients (10.5%; 95% CI, 9.3%-12.0%), whereas noninfectious complications (eg, pain or discomfort, blood in the urine, or sense of urinary urgency) occurred in 1150 patients (55.4%; 95% CI, 53.2%-57.6%) (*P* < .001). Women were more likely to report an infectious complication (92 of 594 [15.5%] women vs 127 of 1482 [8.6%] men; *P* < .001), while men were more likely to report a noninfectious complication (869 of 1482 [58.6%] men vs 281 of 594 [47.3%] women; *P* < .001). Restrictions in activities of daily living (49 of 124 patients [39.5%]) or social activity (54 of 124 [43.9%]) were commonly reported by the patients who had catheters still in place; sexual problems were reported by 99 of 2034 patients (4.9%) after their catheter was removed.

**Conclusions and Relevance:**

Noninfectious complications of urethral catheters affect a substantial number of patients, underscoring the importance of avoiding urethral catheterization whenever possible. Given the high incidence of these patient-reported complications, urethral catheter–associated noninfectious complications should be a focus of surveillance and prevention efforts.

## Key Points

**Question:**

From a patient’s perspective, how common are infectious and noninfectious indwelling urethral catheter–associated complications?

**Findings:**

In this multicenter cohort study of 2076 adults with an indwelling urethral catheter, 57% of patients reported at least 1 complication because of the catheter, and noninfectious complications (55%) were 5 times as common as infectious complications (11%). Women were significantly more likely to report infectious complications, whereas men reported a significantly higher frequency of noninfectious complications.

**Meaning:**

Noninfectious complications involving urethral catheters are common; in addition to avoiding urethral catheterization, patient safety efforts should focus on reducing the noninfectious harms of urethral catheters.

## Teaser

This multicenter cohort study examines infectious and noninfectious complications reported by patients up to a month after receiving an indwelling urethral catheter.

## Introduction

Indwelling urethral catheters are a mainstay in the care of hospitalized patients. An estimated 30 million indwelling urethral (ie, Foley) catheters are sold annually in the United States,^1^ and approximately 20% of hospitalized patients have a urethral catheter at any given time.^2,3^ While indwelling urethral catheters are important for the care of certain hospitalized patients (eg, monitoring hourly urine output in critically ill patients),^4^ they are invasive medical devices and, as such, are a potential threat to patient safety. The most commonly recognized complication is catheter-associated urinary tract infection.^3,5^

Although infectious complications associated with indwelling urethral catheters are well described, few studies have examined the noninfectious complications associated with their use, and Foley catheters lead to several patient safety problems.^6^ For example, a study by Leuck et al^7^ identified 100 instances of Foley catheter–associated trauma in 6513 catheter-days during a period of 16 months. Using administrative data, Aaronson and colleagues^8^ found that urethral catheter–associated complications were linked to an increased length of stay and an increased risk of urinary tract infection among surgical patients.^8^ Finally, Hollingsworth and colleagues^9^ reported in their systematic review of 37 studies that noninfectious urethral catheter–associated complications were as common as clinically significant infections.

Several limitations exist with the previous studies. For example, most were single-site investigations, did not collect data after catheter removal, and often focused on specific patient populations (eg, men, surgical patients, or intensive care unit patients),^7,10,11^ limiting their generalizability. The data sources used are also problematic, with studies using urology consultations likely to overestimate complication rates,^10,11^ whereas those using diagnosis codes^8^ are likely to underestimate complication frequency. Finally, few studies to date have included the perspective of the patient, even though patients can be a reliable source of information about adverse events.^12^ We thus conducted a prospective study to measure the incidence of all complications associated with the indwelling urethral catheter and to capture patient insights about how the use of this device affects their well-being and safety.

## Methods

### Study Overview

We conducted a prospective cohort study at 4 US hospitals in which hospitalized patients with an indwelling urethral catheter were identified and followed up for 30 days after its insertion, even if the catheter had been removed during that time. Data about both infectious and noninfectious catheter–associated complications during this 30-day period were collected through patient interviews. This process included an in-person interview with the patient within 3 days after placement of the catheter and follow-up assessments after 14 days and 30 days, either in-person or through brief telephone interviews. The 4 study sites were the University of Michigan Medical Center and Veterans Affairs Ann Arbor Healthcare System, both in Ann Arbor, Michigan, and Ben Taub Hospital and the Michael E. DeBakey Veterans Affairs Medical Center, both in Houston, Texas. The primary objectives were to determine rates and types of patient-reported infectious and noninfectious complications associated with urethral catheters. Institutional review board approval was received at each participating facility. All participants provided written informed consent.

### Data Collection and Inclusion Criteria

Each weekday morning (Monday through Friday) from August 26, 2015, to August 18, 2017, research staff identified patients on selected inpatient units who, during the previous 3 days, had placement of a urethral catheter. Patient eligibility criteria were (1) hospitalization in an acute care unit, including medical/surgical wards, and intensive care and progressive care units; (2) placement of a urethral catheter for the first time during this hospital stay; (3) the urethral catheter had been in place for no longer than 3 days; (4) patients were at least 18 years of age; and (5) patients were able to speak either English or Spanish. Exclusion criteria were (1) inability to provide self-consent or participate in the interview/assessment process (eg, obtundation, severe dementia, or delirium); (2) refusal to provide written informed consent to participate; or (3) previous participation in this study. After identifying potentially eligible patients by using data from the electronic medical record system, study staff conducted bedside visits to begin the recruitment process. After confirming eligibility, patients were invited to participate regardless of whether the urethral catheter was still in place at the time of recruitment. Once they were recruited, patient follow-up continued for 30 days from the initial date of catheter insertion. Participants received a souvenir magnet as a thank-you gift for their participation.

Information about patient characteristics as well as infectious and noninfectious complications associated with the urethral catheter were collected directly from patients. After providing written consent, an initial in-person patient interview was conducted that included questions about why the urethral catheter was placed. The American Urological Association (AUA) Symptom Index was used to identify any urologic symptom in the previous month (ie, precatheterization).^13^ Follow-up assessments were conducted approximately 14 days and 30 days after catheter insertion. The follow-up assessments, which asked patients about their symptoms and experiences during the previous 2 weeks, were conducted in person if the patient was still hospitalized. After discharge, a study team member contacted the patient by telephone. The patient assessment was designed to elicit the patient’s perspectives about the catheter and to identify complications, such as pain, that may not be well documented in the medical record. Indeed, patient assessments may be the only way to identify complications that occur after hospital discharge, particularly complications that do not lead to a medical visit or require intervention. Questions were primarily closed ended, except for a concluding question that allowed patients to discuss other possible complications.

### Study Measures

Primary outcomes were infectious and noninfectious complication events associated with the urethral catheter. Infectious complications for participants included being told that they had a urinary tract infection or a positive endorsement of any of the following symptoms in the previous 2 weeks: fever, chills, burning with urination, urinary frequency, urinary urgency, or other symptoms suggestive of an infection that required the patient to see a physician. Noninfectious complications for participants whose catheters had been removed included a sense of urgency or bladder spasms, blood in the urine, leaking urine, and difficulty with starting or stopping the urine stream. For those with a catheter still in place, noninfectious complications included pain or discomfort, a sense of urgency or bladder spasms, blood in the urine, and trauma to the skin associated with catheter placement or securement. A full list of complications by category is provided in eTable 1 in the Supplement. Secondary outcomes of interest focused on patient perspectives about their catheters, such as their effect on activities of daily living, social activities, and the general level of comfort.

### Statistical Analysis

We calculated general descriptive statistics for all variables of interest, including baseline patient characteristics (number with percentage or mean [SD]) and outcomes of interest (number with percentage and 95% CI). The primary outcome was the percentage of patients experiencing a complication from a urethral catheter at any time, which was calculated for select individual complications as well as by group (infectious vs noninfectious). The frequency of complications at each site was also assessed. The χ^2^ test was used to compare complication percentages by certain demographic and clinical characteristics. These characteristics included age, sex, indication for catheter placement (perioperative, urinary retention or bladder obstruction, or other), AUA symptom index score prior to catheter use (range, 1-7 [mild]; 8-19 [moderate]; or 20-35 [severe]),^13^ and catheter duration (≤3 days or >3 days). To test whether differences between the sexes persisted after accounting for age, AUA symptom index score, reason for placement, and catheter duration, we used multivariable Poisson regression with robust SEs.^14^

All analyses were performed using SAS, version 9.4 (SAS Institute Inc) or Stata, version 15 (StataCorp). All statistical tests were 2-sided with α set to .05.

## Results

**Figure 1.**
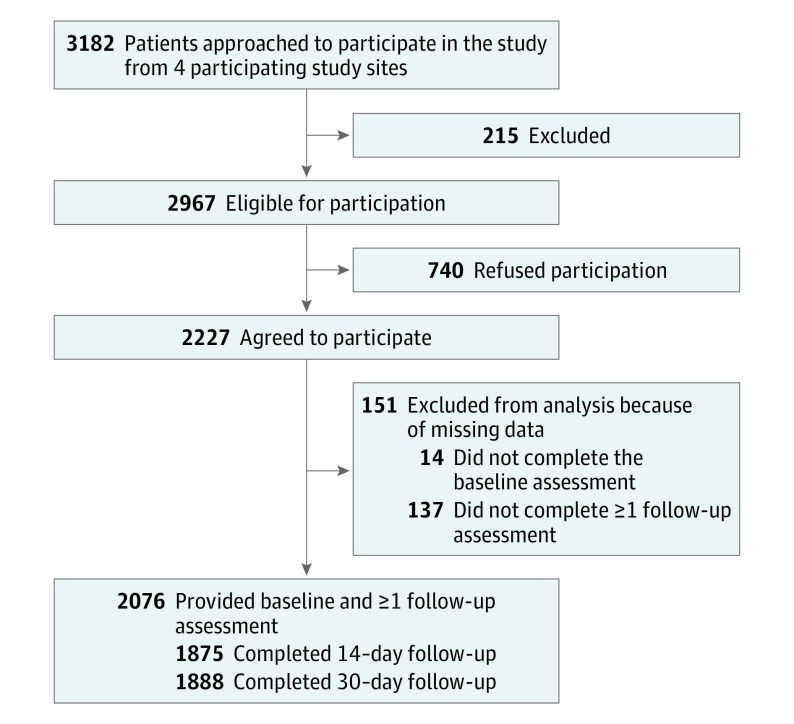
Patient Flowchart of Study Enrollment and Follow-up Of the 2967 patients who met eligibility criteria, 2227 (75.1%) agreed to participate. The 4 participating study sites were the University of Michigan Medical Center and Veterans Affairs Ann Arbor Healthcare System, both in Ann Arbor, Michigan, and Ben Taub Hospital and the Michael E. DeBakey Veterans Affairs Medical Center, both in Houston, Texas.

Of 2967 eligible patients at 4 study sites, 2227 (75.1%) agreed to participate and 2076 patients were evaluated (Figure 1). The majority of patients (1482 of 2076 [71.4%]) were male; mean (SD) age was 60.8 (13.4) years. Participants who were missing a baseline assessment or both follow-up assessments were excluded (151 of 2227 patients [6.8%]), resulting in 2076 patients included in this analysis. Patients were recorded in both the group with a catheter in place and the group with the catheter removed if they had an indwelling catheter at 1 follow-up evaluation, but not at the other follow-up evaluation.

Table 1 describes the patient demographics. Only 594 patients (28.6%) were women because the 2 Veterans Affairs medical centers included in the study provide care for a predominately male population. Urethral catheters were initially placed before surgical procedures in 1653 of 2076 participants (79.6%). Most catheters were placed for short durations, with 1578 patients (76.0%) having them removed within 3 days of insertion. Only 164 patients (7.9%) reported experiencing pain, discomfort, bleeding, or trauma during catheter placement. A significantly lower percentage of patients (33 of 1643 [2.0%]) who had the urethral catheter inserted for a surgical procedure reported complications with insertion, compared with 83 of 144 (57.6%) who had a catheter inserted for urinary retention or bladder obstruction (*P* < .001; eTable 2 in the Supplement). In addition, 414 of 1340 patients (30.9%) whose catheter had been removed prior to enrollment reported experiencing those complications during removal (Table 1).

**Table 1.** Demographic and Baseline Characteristics of 2076 Study Participants

Characteristic	No. (%)
Age, mean (SD), y	60.8 (13.4)
Sex	
Male	1482 (71.4)
Female	594 (28.6)
Race/ethnicity	
White	1631 (78.6)
African American	290 (14.0)
American Indian or Alaskan Native	27 (1.3)
Other or unknown^a^	128 (6.2)
Hispanic	147 (7.1)
Reason for urethral catheter placement	
Perioperative use for surgical procedure	1653 (79.6)
Urinary retention or bladder obstruction	144 (6.9)
Accurate measurement of urine output	30 (1.4)
Patient convenience or incontinence	31 (1.5)
Required prolonged immobilization	25 (1.2)
Other or unknown	193 (9.3)
Experienced pain, discomfort, bleeding, or other trauma during urinary catheter insertion	164 (7.9)
Experienced pain, discomfort, bleeding, or other trauma during urinary catheter removal for participants whose catheter was already removed (n = 1340)	414 (30.9)
Duration of urinary catheter use ≤3 d	1578 (76.0)

^a^ Asian (11 participants [0.5%]), Native Hawaiian or other Pacific Islander (10 [0.5%]), more than 1 race/ethnicity (64 [3.1%]), and unknown (43 [2.1%]).

**Figure 2.**
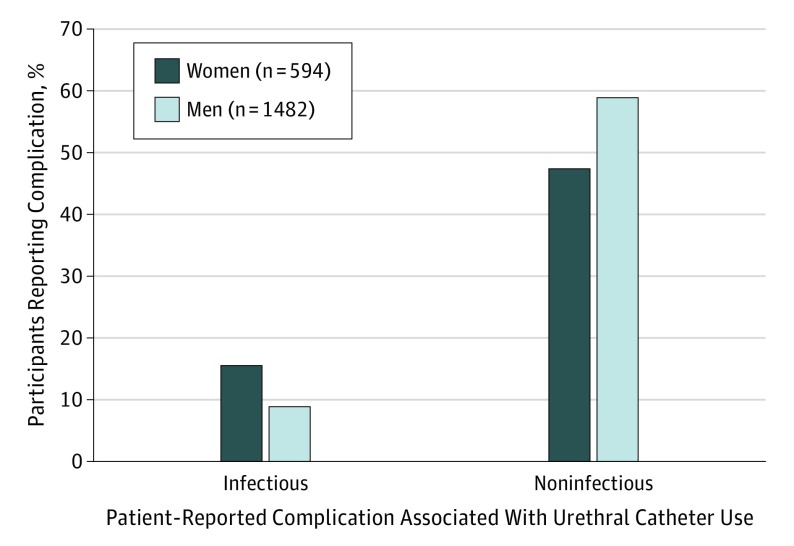
Percentage of 2076 Patients Reporting Infectious or Noninfectious Complications During the Month After Urethral Catheter Insertion Statistically significant differences by sex were found in both infectious and noninfectious patient-reported complications (*P* < .001).

During the 30 days after a urethral catheter was inserted, 1184 of 2076 patients (57.0%; 95% CI, 54.9%-59.2%) reported at least 1 complication because of the indwelling urethral catheter. As shown in Table 2, noninfectious complications were 5 times as prevalent as infectious complications (noninfectious, 55.4%; 95% CI, 53.2%-57.6% vs infectious, 10.5%; 95% CI, 9.3%-12.0%; *P* < .001). Women were more likely than men to report an infectious complication (92 of 594 women [15.5%] vs 127 of 1482 men [8.6%]; *P* < .001), whereas men were more likely than women to report a noninfectious complication (869 of 1482 men [58.6%] vs 281 of 594 women [47.3%]; *P* < .001) as depicted in Figure 2.

**Table 2.** Specific Patient-Reported Complications Associated With Urethral Catheter Use During the Month After Insertion^a^

Specific Complication	No. (%)		
	Catheter in Place (n = 124)	Catheter Removed (n = 2034)	Total (N = 2076)^b^
Infectious complication	19 (15.3)	205 (10.1)	219 (10.5)
Fevers, chills, burning with urination, urinary frequency, urinary urgency, or other symptoms suggestive of an infection that required you to see a physician	12 (9.7)	162 (8.0)	173 (8.3)
Told you have a urinary tract infection	16 (13.0)	106 (5.2)	118 (5.7)
Noninfectious complication	87 (70.2)	1106 (54.4)	1150 (55.4)
Pain or discomfort	67 (54.5)	NA	NA
A sense of urgency or bladder spasms	43 (34.7)	487 (24.0)	523 (25.2)
Blood in the urine	34 (27.4)	179 (8.8)	207 (10.0)
Trauma to your skin related to catheter securement or catheter placement	24 (19.4)	NA	NA
Leaking urine	NA	413 (20.3)	NA
Difficulty with starting or stopping your urine stream	NA	395 (19.5)	NA
Pain or burning when you urinate	NA	353 (17.4)	NA
Split stream of urine	NA	245 (12.1)	NA
Spraying of urine stream	NA	187 (9.2)	NA
Skin problems in the genital area	NA	134 (6.6)	NA
Bleeding from where the urinary catheter entered or was attached to your body, or other type of discharge	NA	94 (4.6)	NA
New urinary tract symptom	NA	69 (3.4)	NA
Bladder or kidney stones	NA	59 (2.9)	NA
Newly diagnosed urethral stricture disease	NA	4 (0.2)	NA
Other complications	66 (53.2)	99 (4.9)	160 (7.7)
Restrictions in activities of daily living associated with having the catheter	49 (39.5)	NA	NA
Restrictions in social activities associated with having the catheter	54 (43.9)	NA	NA
Sexual problems	NA	99 (4.9)	NA
Mechanical or equipment issues with the catheter or securement device, eg, leaking, issues with leg band	16 (12.9)	NA	NA

Abbreviation: NA, not applicable.

^a^ Patients could report more than 1 complication.

^b^ Patients who had a catheter in place at one follow-up evaluation but not the other follow-up evaluation contributed data to both the catheter in place and the catheter removed columns of this table. The column totals do not equal the total sample size.

Table 2 shows the percentage of specific patient-reported complications during the month after urethral catheter insertion. Both infectious and noninfectious complications were reported more frequently by patients who still had their catheter. For 2034 patients who had had their catheter removed, the most frequently cited noninfectious complications were leaking urine (413 of 2034 patients [20.3%]; 95% CI, 18.6%-22.2%), feeling a sense of urgency or bladder spasms (487 [24.0%]; 95% CI, 22.2%-25.9%), and difficulty with starting or stopping the urine stream (395 [19.5%]; 95% CI, 17.8%-21.3%). The most common noninfectious complication cited by the 124 patients who still had a catheter in place was pain or discomfort (67 of 124 patients [54.5%]; 95% CI, 45.3%-63.5%), a sense of urgency or bladder spasms (43 [34.7%]; 95% CI, 26.4%-43.8%), and blood in the urine (34 [27.4%]; 95% CI, 19.8%-36.2%). In addition to infectious and noninfectious complications, many of the 124 patients who still had a catheter reported activities of daily living restrictions (49 of 124 [39.5%]; 95% CI, 30.9%-48.7%) and social activity restrictions (54 of 124 [43.9%]; 95% CI, 35.0%-53.1%). Of the 124 patients, 16 (12.9%) with a catheter in place reported mechanical or equipment issues with the catheter or securement device. Sexual problems were reported by 99 of 2034 patients (4.9%) who had a catheter that had been removed (95% CI, 4.0%-5.9%).

Select clinical characteristics associated with patient-reported infectious and noninfectious complications are shown in Table 3. For example, a higher percentage of patients with a baseline AUA symptom index score in the severe range reported both infectious complications (37 of 213 patients [17.4%]; *P* < .001) and noninfectious complications (175 of 213 [82.2%]; *P* < .001) than those with mild or moderate scores (mild score: infectious, 98 of 1176 [8.3%] and noninfectious, 519 [44.1%]; moderate score: infectious, 79 of 670 [11.8%] and noninfectious, 442 [66.0%]). Longer duration of urethral catheter use was also associated with more reported infectious and noninfectious complications. Medical or surgical indication for catheter placement was not significantly associated with infectious complications. Noninfectious complications, however, were reported by a higher percentage of patients with a catheter inserted due to urinary retention or bladder obstruction. In a multivariable analysis adjusting for demographic and clinical characteristics (eTable 3 in the Supplement), the sex-related difference remained significant only for infectious complications (incident rate ratio [IRR] for female patients, 2.11; 95% CI, 1.59-2.79; *P* < .001). Moderate and severe AUA symptom index scores and urinary catheter duration of more than 3 days remained significantly associated with both infectious and noninfectious complications (eTable 3 in the Supplement).

**Table 3.** Clinical Characteristics Associated With Infectious and Noninfectious Indwelling Urethral Catheter Complications

Clinical Characteristic	Infectious Complication, No. (%)	*P* Value	Noninfectious Complication, No. (%)	*P* Value
Age, mean (SD), y	60.7 (13.6)^a^	.87	62.4 (13.0)^b^	<.001
Reason for urethral catheter placement, No. (%)				
Perioperative use for surgical procedure (n = 1653)	174 (10.5)	.29	880 (53.2)	<.001
Urinary retention or bladder obstruction (n = 144)	20 (13.9)		104 (72.2)	
Other or unknown (n = 279)	25 (9.0)		166 (59.5)	
AUA Symptom Index score, No. (%)				
Mild (n = 1176)	98 (8.3)	<.001	519 (44.1)	<.001
Moderate (n = 670)	79 (11.8)		442 (66.0)	
Severe (n = 213)	37 (17.4)		175 (82.2)	
Duration of urinary catheter, No. (%)				
≤3 d (n = 1578)	154 (9.8)	.04	796 (50.4)	<.001
>3 d (n = 498)	65 (13.1)		354 (71.1)	

Abbreviation: AUA, American Urological Association.

^a^ Comparison group: no infectious complications; mean (SD) age, 60.8 (13.4) years.

^b^ Comparison group: no noninfectious complications; mean (SD) age, 58.8 (13.8) years.

## Discussion

This prospective cohort study of urethral catheter–associated complications conducted at 4 US medical centers in 2 states has 3 key findings. First, the overall patient-reported complication rate of indwelling urethral catheter use was found to be 57.0%, higher than what is commonly reported in the literature.^7,9^ Second, noninfectious complications were reported 5 times more often than infectious complications. However, women were almost twice as likely to report an infectious complication (15.5% vs 8.6%), while men were more likely to report a noninfectious complication (58.6% vs 47.3%). Finally, more than one-third of patients with catheters in place reported restrictions in activities of daily living (39.5%) and social activity (43.9%). These findings are novel, relevant to patient safety, and could not have been uncovered without direct follow-up in our study of patients who had been catheterized.

Other investigators have also evaluated the noninfectious complications of urethral catheters, albeit without the direct patient perspective. For example, Leuck et al^7^ prospectively reviewed the medical records at a single Veterans Affairs hospital to evaluate both the infectious and traumatic complications associated with the use of an indwelling urethral catheter. When reports of traumatic episodes associated with pain or that required intervention were compared with episodes of symptomatic urinary tract infection (the Centers for Disease Control and Prevention’s current focus for surveillance and treatment), trauma was significantly more common than infection (0.9 per 100 catheter-days vs 0.3 per 100 catheter-days; *P* < .001).^7^ Using administrative data on surgical procedures, Aaronson et al^8^ concluded that catheter-associated complications were reported rarely but were significantly associated with increased length of hospital stay and catheter-associated urinary tract infection rates. Awad and colleagues^15^ conducted a legal database review of the association of urethral catheters with medical malpractice from 1965 to 2015 and reported that the most common malpractice claim was traumatic insertion of a urethral catheter (48% of claims). These investigators noted that several of the lawsuits involved a spouse who claimed loss of consortium (deprivation of the benefits of a family relationship because of injuries caused by the tort). Finally, Davis and colleagues^16^ followed up patients at 2 tertiary care hospitals in Ireland for 6 months and reported an incidence of traumatic urethral catheterization of 6.7 per 1000 catheters with an additional length of hospital stay of more than 9 days for those patients coupled with incremental health care costs.

Our findings confirm the importance of noninfectious complications based on reports of a diverse group of patients who received care at 4 different medical centers. We also received patient-reported accounts about their experience with the use of the catheter. Many patients complained about the indwelling catheter. For example, one patient reported, “I never want another Foley catheter. Hurts like hell!” Another reported that the catheter was “uncomfortable, cumbersome, and difficult to sleep with.” Many patients noted that the removal process was very painful: “It felt like it was cutting me coming out.” Not all patients reported negative personal experiences with the indwelling catheter. A few patients contacted viewed the urethral catheter favorably: “It was my first experience with a catheter; it wasn’t too bad.”

We prospectively followed up a large cohort to identify complications that resulted from urethral catheterization and were described by patients rather than relying on information that may be documented in the medical record or may be available through secondary data sources. This information provided a unique and important perspective on potential complications that may concern patients and may occur outside the hospital setting. For example, urine leaking from the catheter when the device was in place or from the urethra after it had been removed was an issue for many patients. As one patient stated, “I wasn’t given any instruction on how to handle a leak”; this incident resulted in his researching information on the internet. Our study extended understanding of urethral catheter–associated complications by also identifying lifestyle issues—such as sexual problems—that are important to patients. In the words of one patient, “I am mostly dissatisfied with my urinary condition because I have not been able to have sex in a very long time.”

The analysis also identified sex-related differences in reported complications, which makes some sense given the anatomical differences of the genitourinary tracts. Women are at higher risk for catheter-associated urinary tract infections than men because they have shorter urethras and closer proximity of perineal bacterial colonization to the insertion site of the indwelling catheter.^17^ For male patients, complications potentially associated with the presence of an enlarged prostate gland (eg, urinary retention) were seen more commonly than the same complications in women. Although male sex was no longer an independent risk factor for noninfectious complications when assessed in a multivariable model including the AUA symptom index score, this is likely linked to collinearity with the AUA symptom index score.

Our findings have clinical implications. We should launch efforts to reduce the noninfectious complications of urethral catheters in the same way that we have used collaborative efforts to reduce catheter-associated urinary tract infections in some patient populations.^18,19^ Attention to the technical aspects of proper catheter insertion and maintenance as well as increased focus on reducing unnecessary catheter insertions and unnecessary days of catheterization duration seem likely to reduce noninfectious and infectious complications. As Meddings and colleagues^20^ concluded in a systematic review of 30 studies, reminders about an indwelling urinary catheter and stop orders should be used to improve patient safety because these approaches will reduce both types of catheter complications. The indwelling urethral catheter has even been referred to as a “1-point restraint,”^21^ which underscores the importance of limiting the use of catheters. Strategies for better tracking and surveillance of noninfectious complications, such as major trauma or bleeding, should also be considered. Incorporating what happens after the hospital stay may fully capture possible complications of this invasive procedure.

### Limitations

This study should be interpreted in the context of the following limitations. We conducted this study in the United States at 4 sites within 2 states, and we included only patients who received an indwelling catheter during acute care hospitalization. Thus, the findings may not be generalizable to all patients who receive a urethral catheter, such as those who are evaluated in the emergency department for urinary retention and discharged to home. We might expect worse outcomes in these patients; they often have difficulty obtaining timely follow-up care for removal of the catheter. Also, one of the sites contributed modestly to the overall number of patients enrolled for reasons primarily related to a population of younger patients who had a low baseline need for indwelling urethral catheters. In addition, the information was collected directly from the patient. Although patient-reported complications may not constitute a medically defined complication because of well-known poor documentation of urethral catheter complications,^22^ we believe that what patients report is important to understand the full scope of potential problems. Without an approach to validate their reports, however, inclusion of patient-reported complications could result in an overestimate of true medical complications.

## Conclusions

We provided important estimates of the burden of complications due to the use of this device. We also provided a foundation for interventional studies to further ameliorate complications associated with catheter use. Urethral catheters, which are often used to help safely manage care of the patient during hospitalization, often lead to the opposite result. In light of the frequency with which urethral catheters are used, we should consider not only infectious complications but also the noninfectious complications associated with these catheters as key areas of possible harms and thus vital targets for future prevention efforts.

## Notes

Supplement. **eTable 1.** Categorization of Urethral Catheter–Associated Complications **eTable 2.** Reason for Catheter Placement and Reported Experience During Catheter Insertion **eTable 3.** Multivariable Linear Regression Model to Predict Infectious and Noninfectious Urethral Catheter Complications

## References

[1] Elder M US catheter market In: Global Markets for Catheters. Wellesley, MA: BCC Research; 2016:91-96.

[2] Saint S Clinical and economic consequences of nosocomial catheter-related bacteriuria. Am J Infect Control. 2000;28(1):68-75. doi:10.1016/S0196-6553(00)90015-4 10679141

[3] Magill SS, Edwards JR, Bamberg W, ; Emerging Infections Program Healthcare-Associated Infections and Antimicrobial Use Prevalence Survey Team Multistate point-prevalence survey of health care–associated infections. N Engl J Med. 2014;370(13):1198-1208. doi:10.1056/NEJMoa1306801 24670166 PMC4648343

[4] Meddings J, Saint S, Fowler KE, The Ann Arbor Criteria for appropriate urinary catheter use in hospitalized medical patients: results obtained by using the RAND/UCLA appropriateness method. Ann Intern Med. 2015;162(9)(suppl):S1-S34. doi:10.7326/M14-1304 25938928

[5] World Health Organization Report on the Burden of Endemic Health Care–Associated Infection Worldwide: A Systematic Review of the Literature. Geneva, Switzerland: World Health Organization; 2011.

[6] Fakih MG, Gould CV, Trautner BW, Beyond infection: device utilization ratio as a performance measure for urinary catheter harm. Infect Control Hosp Epidemiol. 2016;37(3):327-333. doi:10.1017/ice.2015.287 26894622 PMC6502466

[7] Leuck AM, Wright D, Ellingson L, Kraemer L, Kuskowski MA, Johnson JR Complications of Foley catheters—is infection the greatest risk? J Urol. 2012;187(5):1662-1666. doi:10.1016/j.juro.2011.12.113 22425122

[8] Aaronson DS, Wu AK, Blaschko SD, McAninch JW, Garcia M National incidence and impact of noninfectious urethral catheter related complications on the Surgical Care Improvement Project. J Urol. 2011;185(5):1756-1760. doi:10.1016/j.juro.2010.12.041 21420117

[9] Hollingsworth JM, Rogers MA, Krein SL, Determining the noninfectious complications of indwelling urethral catheters: a systematic review and meta-analysis. Ann Intern Med. 2013;159(6):401-410. doi:10.7326/0003-4819-159-6-201309170-00006 24042368

[10] Kashefi C, Messer K, Barden R, Sexton C, Parsons JK Incidence and prevention of iatrogenic urethral injuries. J Urol. 2008;179(6):2254-2257. doi:10.1016/j.juro.2008.01.108 18423712

[11] Thomas AZ, Giri SK, Meagher D, Creagh T Avoidable iatrogenic complications of urethral catheterization and inadequate intern training in a tertiary-care teaching hospital. BJU Int. 2009;104(8):1109-1112. doi:10.1111/j.1464-410X.2009.08494.x 19338562

[12] Zhu J, Stuver SO, Epstein AM, Schneider EC, Weissman JS, Weingart SN Can we rely on patients’ reports of adverse events? Med Care. 2011;49(10):948-955. doi:10.1097/MLR.0b013e31822047a8 21642876

[13] Barry MJ, Fowler FJ Jr, O’Leary MP, ; Measurement Committee of the American Urological Association The American Urological Association symptom index for benign prostatic hyperplasia. J Urol. 1992;148(5):1549-1557. doi:10.1016/S0022-5347(17)36966-5 1279218

[14] Zou G A modified poisson regression approach to prospective studies with binary data. Am J Epidemiol. 2004;159(7):702-706. doi:10.1093/aje/kwh090 15033648

[15] Awad MA, Osterberg EC, Chang H, Urethral catheters and medical malpractice: a legal database review from 1965 to 2015. Transl Androl Urol. 2016;5(5):762-773. doi:10.21037/tau.2016.08.02 27785434 PMC5071201

[16] Davis NF, Quinlan MR, Bhatt NR, Incidence, cost, complications and clinical outcomes of iatrogenic urethral catheterization injuries: a prospective multi-institutional study. J Urol. 2016;196(5):1473-1477. doi:10.1016/j.juro.2016.05.114 27317985

[17] Maki DG, Tambyah PA Engineering out the risk for infection with urinary catheters. Emerg Infect Dis. 2001;7(2):342-347. doi:10.3201/eid0702.010240 11294737 PMC2631699

[18] Saint S, Greene MT, Krein SL, A program to prevent catheter-associated urinary tract infection in acute care. N Engl J Med. 2016;374(22):2111-2119. doi:10.1056/NEJMoa1504906 27248619 PMC9661888

[19] Saint S, Fowler KE, Sermak K, Introducing the No Preventable Harms Campaign: creating the safest health care system in the world, starting with catheter-associated urinary tract infection prevention. Am J Infect Control. 2015;43(3):254-259. doi:10.1016/j.ajic.2014.11.016 25728151

[20] Meddings J, Rogers MA, Krein SL, Fakih MG, Olmsted RN, Saint S Reducing unnecessary urinary catheter use and other strategies to prevent catheter-associated urinary tract infection: an integrative review. BMJ Qual Saf. 2014;23(4):277-289. doi:10.1136/bmjqs-2012-001774 24077850 PMC3960353

[21] Saint S, Lipsky BA, Goold SD Indwelling urinary catheters: a one-point restraint? Ann Intern Med. 2002;137(2):125-127. doi:10.7326/0003-4819-137-2-200207160-00012 12118969

[22] Meddings JA, Reichert H, Rogers MA, Saint S, Stephansky J, McMahon LF Effect of nonpayment for hospital-acquired, catheter-associated urinary tract infection: a statewide analysis. Ann Intern Med. 2012;157(5):305-312. doi:10.7326/0003-4819-157-5-201209040-00003 22944872 PMC3652618

