# Supplementary material for: A Multicenter Study of Patient-Reported Infectious and Noninfectious Complications Associated With Indwelling Urethral Catheters
Source: JAMA Intern Med. 2018 Jul 2;178(8):1078–85. doi: 10.1001/jamainternmed.2018.2417 (PMC6143107; doi:10.1001/jamainternmed.2018.2417)
Supplement: Supplement. — eTable 1. Categorization of Urethral Catheter–Associated Complications eTable 2. Reason for Catheter Placement and Reported Experience During Catheter Insertion eTable 3. Multivariable Linear Regression Model to Predict Infectious and Noninfectious Urethral Catheter Complications [file jamainternmed-178-1078-s001.pdf]

Supplementary Online Content

Saint S, Trautner BW, Fowler KE, et al. A multicenter study of patient-reported infectious and noninfectious complications associated with indwelling urethral catheters. *JAMA Intern Med.* Published online July 2, 2018. doi:10.1001/jamainternmed.2018.2417

eTable 1. Categorization of Urethral Catheter–Associated Complications

eTable 2. Reason for Catheter Placement and Reported Experience During Catheter Insertion

eTable 3. Multivariable Linear Regression Model to Predict Infectious and Noninfectious Urethral Catheter Complications

This supplementary material was provided by the authors to give readers further details on their article.

© 2018 Saint S et al. *JAMA Internal Medicine.*

eTable 1. Categorization of Urethral Catheter–Associated Complications

	Patient-Reported Complication	
**Categorization**	**Catheter in Place**	**Catheter Removed**
Infectious	• Fever, chills, burning with urination, urinary frequency, urgency, or other symptoms suggestive of an infection that required them to see a doctor<br>• Were told they had a urinary tract infection	• Fever, chills, burning with urination, urinary frequency, urgency, or other symptoms suggestive of an infection that required them to see a doctor<br>• Were told they had a urinary tract infection
Non-Infectious	• A sense of urgency or bladder spasms<br>• Blood in urine<br>• Pain or discomfort<br>• Trauma to skin (related to securement or catheter placement)	• A sense of urgency or bladder spasms<br>• Bladder/kidney stones<br>• Bleeding from where the urinary catheter entered or was attached to body, or other discharge<br>• Blood in urine<br>• Difficulty with starting or stopping urine stream<br>• Leaking urine<br>• Newly diagnosed urethral stricture disease<br>• New urinary tract symptoms<br>• Pain or burning when urinating<br>• Skin problems in the genital area<br>• Split stream of urine<br>• Spraying of urinary stream

© 2018 Saint S et al. *JAMA Internal Medicine.*

eTable 2. Reason for Catheter Placement and Reported Experience During Catheter Insertion

	Experienced pain, discomfort, bleeding, or other trauma during urinary catheter insertion	*P* Value
Reason for Urethral Catheter Placement, No. (%)		
Perioperative use for surgical procedure (n=1643)	33 (2.0)	<.001
Urinary retention or bladder obstruction (n=144)	83 (57.6)	
Other or unknown (n=276)	48 (17.4)	

© 2018 Saint S et al. *JAMA Internal Medicine.*

eTable3. Multivariable Linear Regression Model to Predict Infectious and Noninfectious Urethral Catheter Complications

	Infectious Complication		Non-Infectious Complication	
Variable	IRR (95% CI)	P Value	IIR (95% CI)	P Value
Age (10 years)	1.02 (0.92-1.13)	.70	1.04 (1.01-1.07)	.02
Sex				
Male	1 [Reference]		1 [Reference]	
Female	2.11 (1.59-2.79)	<.001	0.93 (0.84-1.02)	.14
AUA Symptom Index Score				
Mild	1 [Reference]		1 [Reference]	
Moderate	1.46 (1.10-1.95)	.01	1.41 (1.30-1.54)	<.001
Severe	2.26 (1.56-3.26)	<.001	1.66 (1.51-1.83)	<.001
Reason for Urethral Catheter Placement				
Perioperative use for surgical procedure	1 [Reference]		1 [Reference]	
Urinary retention or bladder obstruction	1.20 (0.75-1.91)	.44	1.10 (0.98-1.24)	.11
Other or unknown	0.83 (0.56-1.23)	.35	1.05 (0.95-1.17)	.36
Urinary Catheter Duration				
3 days or Less	1 [Reference]		1 [Reference]	
More than 3 days	1.38 (1.03-1.84)	.03	1.27 (1.17-1.37)	<.001

Abbreviations: IRR, Incident Rate Ratio; CI, Confidence Interval; AUA, American Urological Association

© 2018 Saint S et al. *JAMA Internal Medicine.*
